# What is the effectiveness of a personalised video story after an online diabetes risk assessment? A Randomised Controlled Trial

**DOI:** 10.1371/journal.pone.0264749

**Published:** 2022-03-03

**Authors:** Susan L. Williams, Quyen To, Corneel Vandelanotte

**Affiliations:** Central Queensland University, School of Health Medical and Applied Sciences, Physical Activity Research Group, Appleton Institute, Queensland, Australia; Weill Cornell Medical College in Qatar, QATAR

## Abstract

**Background:**

Online risk assessment tools for type 2 diabetes communicate risk information to motivate individuals to take actions and reduce their risk if needed. The impact of these tools on follow-up behaviours (e.g., General Practitioner (GP) visits, improvement in health behaviours) is unknown. This study examined effectiveness of a personalised video story and text-based message on GP and health professional visitations and health behaviours, of individuals assessed as ‘high risk’ following completion of the online Australian Type 2 Diabetes Risk Assessment Tool (AUSDRISK).

**Methods:**

A Randomised Controlled Trial (conducted between October 2018 and April 2019) included 477 participants with a high score (≥12). The control group received a text-based message (TM) and the intervention group received both the text-based message and a personalised video story (TM+VS) encouraging them to take follow-up action. Participants reported follow-up actions (one- and three months), and physical activity (PA), dietary behaviours and body weight (baseline, one and three months). Generalized Linear Mixed Models and chi-squared tests were used to test differences in outcomes between groups over time.

**Results:**

The intervention was not more effective for the TM+VS group compared to the TM only group (p-values>0.05 for all outcomes). More participants in the TM only group (49.8% compared to 40.0% in the VS+TM group) visited either a GP or health professional (*p* = 0.18). During the 3-month follow-up: 44.9% of all participants visited a GP (36.7%) and/or other health professional (31.0%). Significant improvements were found between baseline and three months, in both groups for weekly physical activity, daily fruit and vegetable intake and weight status.

**Conclusions:**

Messages provided with online diabetes risk assessment tools to those with high-risk, positively influence GP and health professional visitations and promote short-term improvements in health behaviours that may contribute to an overall reduction in the development of type 2 diabetes.

**Trial registration:**

Australia New Zealand Clinical Trials Registry; ACTRN12619000809134.

## Introduction

Globally, type 2 diabetes affects more than 9% of adults and causes more than four million deaths [1]. Up to 50% of cases of type 2 diabetes are considered preventable if lifestyle related risk factors are managed by, for example, maintaining a healthy body weight, being physically active and eating a healthy diet [1, 2]. Type 2 diabetes has a long latent phase, and many people are asymptomatic for many years prior to diagnosis [3]. Early detection is considered important to identify individuals with pre-diabetes (impaired glucose tolerance) and undiagnosed type 2 diabetes, to delay onset of the disease (in the case of pre-diabetes), prevent long-term complications, increase life expectancy, and reduce lifetime costs [4–7]. Lifestyle interventions to improve diet and physical activity can also limit or delay progression of diabetes [8].

Non-invasive diabetes risk assessment tools are inexpensive and easy to administer and aim to provide preliminary assessment of population groups for risk of type 2 diabetes [9] and identify individuals who may benefit from programs that address poor lifestyle behaviours [10]. The premise of online personalised risk calculators is that communicating individualised health risk information will motivate and guide individuals to understand and ultimately take actions to reduce their risk [11]. Relatively few studies have investigated or reported overall use of these tools by the general population, however some evidence from a systematic review of how these tools are used in practice suggests lower use by males, younger ages, those who are socially or geographically isolated, low skilled or unemployed, middle to high income earners and those at high risk of type 2 diabetes [12]. Low use in the general population through self-administration may relate to lack of perceived risk, fear of complexity of the test, fear of the disease and uncertainty about where to go if a high-risk score is calculated [13].

The Australian Type 2 Diabetes Risk Assessment Tool (AUSDRISK) is designed for use in Australia by individuals or health professionals to assist in predicting five-year risk of diabetes [14]. Similar to the FINDRISC [10], the AUSDRISK includes 10 weighted points-based questions relating to known risk factors for type 2 diabetes (age, gender, ethnicity, family history of the disease, blood pressure medication, cigarette smoking, fruit and vegetable intakes, physical activity and waist circumference/weight status) and responses to the questions permit calculation of an individual’s risk of developing type 2 diabetes in the next five years (either: low; intermediate; or high risk). The AUSDRISK has been validated as a reliable assessment tool for Australian adults [14] and its utility for identifying risk of type 2 diabetes has been found to be reasonable to high [15, 16].

The AUSDRISK is commonly available as an online self-assessment tool and once completed, individuals receive their risk score and a static text-based message (either directly on a website or via e-mail) that outlines what their risk score means and ‘what to do next to prevent diabetes’. The AUSDRISK is however, underutilised in clinical practice [17] and very little is known regarding use through self-administration via organisational websites and the impact of calculated risk scores on follow-up behaviours of individuals who receive a high-risk score. According to a 2014/15 Diabetes Queensland Risk Assessment Annual Report, of the 91 survey respondents who had received a moderate or high-risk score, 36% followed up with their doctor to discuss their risk and a further 10% were planning to do so [18].

Although, research of diabetes risk assessment tools and their impact is relatively limited, studies of such tools for other health conditions (e.g., cardiovascular disease) and behaviours (e.g., physical activity) suggest that providing risk information alone or in combination with education, increases perceived risk and intention to modify behaviours, though impact may be dependent upon repetition of messages and risk information overtime [19]. Text messaging interventions may improve health behaviours in the short term (up to 12 weeks) [20] however, compared to text-based information (which is usually skimmed and scanned with limited in-depth reading), video-based information may be more effective as it is more relatable [21] and less likely dismissed [22, 23], attracts more attention [24, 25] and when included in intervention programs can be more effective in motivating behaviour change [21, 25, 26]. Whilst there is strong evidence that personalisation/tailoring of risk information on its own is ineffective in creating sustainable behaviour change [27] some research has found that providing a combination of written and tailored video messages can result in sustainable change in dietary behaviours [28].

With few previous studies conducted of online diabetes risk assessment tools and little knowledge of their impact on follow-up behaviours of individuals who receive a high-risk score, we developed the Diabetes Online Risk Assessment (DORA) study. The study design was based on previous evidence for the effectiveness of using an audio-visual format to communicate health information and increase awareness of health conditions [29] and combining factual information with a human-interest perspective to make health messages easier to comprehend and more engaging [30].

### Specific objectives or hypotheses

This paper reports the primary outcomes of the DORA study. Specifically, the impact of a static text-based message versus the combination of this text-based message and a personalised video story, on the follow-up behaviours of adults who received a high-risk score following completion of the online AUSDRISK. We hypothesised that the intervention effect (follow-up with a medical professional, changes in health status and changes in health behaviours [dietary intake, physical activity]), would be significantly higher when a high diabetes risk score is presented as a combination of a text-message with a video-based story (VS+TM) compared to text-based message only (TM).

## Materials and methods

### Trial design and changes to methods after trial commencement

This was a parallel-group, active-controlled trial with block randomisation of adults with a high AUSDRISK score (≥12), conducted in Australia. Changes were made to the original protocol but before recruitment of any participants. These changes included: (i) extension of participant recruitment dates, (ii) recruitment of only adults with a high-risk (score ≥12) (rather than those with intermediate (score 9–11) or high-risk score), and (iii) inclusion of self-reported height and body weight measures at three-month follow-up. These modifications were made to account for (i) delays experienced in development of the study website, (ii) to refine the study sample and promote recruitment of individuals most likely to have undiagnosed type 2 diabetes and who would benefit most from follow-up consultation and advice (i.e., those with high risk) [4, 31], and (iii) to measure any change in weight status they may have occurred as a result of behaviour change following receipt of their risk score and message.

This Randomised Control Trial was registered with the Australian New Zealand Clinical Trials Registry in June 2019 (ACTRN12619000809134) and data collection commenced October 10, 2018 and was completed April 7, 2019. Ethical approval to conduct the study was granted by the Central Queensland University Human Research and Ethics Committee (HREC) (H17/10-176) in February 2018. Approval was also provided by the HREC in August 2018, for changes made to the original protocol (as outlined above). The authors confirm that all ongoing and related trials for this intervention are registered and that retrospective registration of the study (after enrolment of the first participants) occurred due to an administrative error, and there were no changes to primary outcome measures or study protocols between ethical approval and trial registration.

### Participant eligibility criteria, setting and location

We used online methods (Facebook sites, community websites and email systems) to invite participants. From the invitation, individuals were provided a link to the DORA study website where they were given full study information and asked to complete initial online screening questions related to the eligibility criteria: aged over 35 years, living in Australia, access to a reliable internet connection, ability to comfortably speak and read the English language, no known diagnosis with type 2 diabetes. Explicit consent to participate in the study (and including consent for data publication) was sought from eligible individuals prior to their completion of any study survey. To obtain consent, prior to commencing each survey on the study website, participants were provided with an outline of study processes and a link to a complete study information sheet. Participants were then asked to click on YES on the front webpage of the study website to indicate that they had read and understood all study information and consented to their involvement in the study. All consenting participants who were also eligible based on screening, were then provided with a username and password to provide secure and ongoing access to the study website (and study resources) for the duration of the study.

All consenting participants completed the online AUSDRISK questions and those with a score of 11 points or less (low- or intermediate-risk) were provided with a message that advised them of their score and basic information about what their score means in relation to their risk of developing type 2 diabetes (similar to information provided on other diabetes web pages [32] and that based on their score they were not eligible to participate in the full study. Participants with an intermediate-risk score were also provided with a downloadable letter outlining their risk score and how the score was obtained, that could be taken to their doctor if the individual chose to seek additional advice.

Participants with a high-risk score were asked to complete online, a baseline survey and one- and three-month follow-up surveys. Email reminders (maximum of 3) were automatically sent to each participant 30- and 90-days following completion of their baseline survey. Participants were provided with a $20 (AUD) shopping voucher upon completion of each of the one- and three-month surveys.

### Interventions

All participants allocated to the control (TM) group were provided—via the study website, with a static text-based message only (See S1 File) and a downloadable letter for their doctor providing explanation of their diabetes risk score and how the score was obtained. An option to print their text-based message was also provided. The text-based message included explanation of their risk score and factors known to increase risk of type 2 diabetes and advice on what to do next. Each participant allocated to the intervention group (VS+TM) received via the study website, a video-based story that ranged from between 80 and 144 seconds in duration. From the study website, participants could download their video story or access the video for replay at any time.

Based on the Health Belief Model [33] we tailored the video-based stories to specific individual risk factors for type 2 diabetes. A total of 13 video-based stories (with broad scripts) were developed. Each video-based story was tailored by gender, age and risk factors included in the AUSDRISK including body weight status, previous gestational diabetes (females only), family history of type 2 diabetes, low intakes of fruit and vegetables, low physical activity levels. Six actors/actresses were employed to deliver the scripted video-based stories for different scenarios that were developed in relation to different risk profiles. The physical appearances of the actors/actresses were altered with make-up and clothing to visually represent different age groups and body shape/weight status (healthy weight or overweight/obese). Each video-based story portrayed an individual diagnosed with type 2 diabetes and their personal history (e.g., gestational diabetes, family history of type 2 diabetes) and journey from diagnosis to treatment and related experiences. In each of the video-based stories, an emphasis was placed on early detection through follow-up visits with their General Practitioner (GP) for further testing and the health risks associated with type 2 diabetes; development of the disease; why early detection is beneficial; the procedure for follow-up, and further testing and possible outcomes of any subsequent test.

The DORA study website used IF-THEN decision rules for the allocation of videos based on participant responses to questions in the AUSDRISK (for example; if a female participant was over 55 years of age and had previous history of gestational diabetes and did not do at least 2.5 hours of physical activity per week (risk score 14), this participant would have received a video that was presented by female who appeared to be of similar age and included discussion of her experience of gestational diabetes and her low physical activity levels).

To support individuals in seeking further information or advice regarding healthy lifestyle behaviours after receipt of their risk score, the study website included additional links to reputable information sources including national and state-wide diabetes organisations, nutrition organisations (e.g. Nutrition Australia, Dietitians Association of Australia), government health websites and online health programs that encourage healthy lifestyle behaviours (e.g., 10,000 Steps, My Health for Life, Heart Foundation, Get Healthy Coaching).

### Outcome measures

The outcome measures relate to the follow-up actions of participants in visiting their GP (primary) and other healthcare professionals (secondary). In the one- and three-month follow-up surveys, participants were asked two questions relating to their follow-up behaviours: (i) ‘Did the video/text information you received after the DORA baseline survey motivate you to seek further advice from your doctor/GP information’ and (ii) ‘Since receiving your risk rating have you sought further advice from any of the following health professionals (Dietitian, Nutritionist, Naturopath, Exercise Consultant, Other)’ Participants who responded ‘Yes’ to either of these questions at any timepoint between baseline and three-months, were categorised as taking follow-up action with (i) their GP and/or (ii) other health professionals. Those who answered ‘no’ were also asked to report any reasons for not seeking further advice from their GP or other health professionals.

#### Socio-demographic and health characteristics

In addition to socio-demographic data collected via questions included in the AUSDRISK (age, gender, ethnicity), the baseline survey included questions to capture characteristics relating to the individuals marital status (Partnered/Unpartnered); highest level of education (year 12 or less, Technical and Further Education;//trade studies/Tertiary education and above); Current employment status (Fulltime/Parttime and Casual/Unemployed); Gross annual household income (High (>$2500 AUD/week)/ Middle ($1000-$2499AUD/week)/Low (<$1,000 AUD/week); and Geographical living location (Major city/Inner Regional/Outer Regional/Rural or Remote).

The baseline and three- month follow-up surveys also asked participants to self-report their height (in centimetres) and body weight (in kilograms) to permit calculation of their Body Mass Index (BMI) and examination of any change in weight status from baseline to three-months. These measures were not collected at the one-month follow-up as it was not expected that a significant change in BMI would be detected within four weeks without a lifestyle intervention being implemented in addition to risk messaging [34]. Categories for weight status were created in accordance with standardised cut-offs [35] as: Healthy weight (BMI <24.99kg/m^2^); Overweight (BMI 25–29.99 kg/m^2^); or Obese (BMI 30 kg/m^2^).

eHealth literacy was also measured at baseline using the validated eight-item eHEALS measure of an individual’s combined knowledge, comfort, and perceived skills at finding, evaluating, and applying electronic health information to health problems. This measure uses a 5-point Likert scale (1-strongly disagree, 5-strongly agree), and the score ranges from 8 to 40, with a higher score indicating higher literacy [36, 37].

#### Health behaviours

The Active Australia Survey [38] which has shown reliability and validity in middle aged women [39] and less active adults [40], was used to measure participation in leisure-time physical activity. In accordance with recommendations for calculations of total weekly physical activity time, total activity sessions and categorisations for physical activity [38], participants were categorised as either: Sufficiently active (≥150 minutes of MVPA for ≥5 sessions/week); or Insufficiently active (<150 minutes or >150 minutes and < 5 sessions).

A short, validated Food Frequency Questionnaire (FFQ) [41] was used to measure dietary intake behaviours. Participants reported their intakes of a range of foods from each food group over the past month. Daily fruit (fresh, dried and juices) and vegetable intakes (fresh/raw, canned, dried, cooked) were calculated at baseline, and one- and three-month follow-ups. In accordance with author guidelines for the FFQ, monthly intakes were converted to daily intakes. Daily intakes were summed and in accordance with the Australian Dietary Guidelines for daily intakes of fruit (two or more serves/day) and vegetables (five or more serves/day) [42] participants were subsequently categorised as either (i) not meeting or (ii) meeting guidelines for fruit and vegetables.

Additional measures of diabetes symptoms; intentions to change-diet and physical activity, weight status; perceived risk of diabetes; need for cognition, and acceptability of message, were included in the DORA study. Outcomes related to these measures are not included in this paper as they are beyond the scope of reporting primary outcomes of the study. No changes to trial outcomes were made after trial commencement.

### Sample size

The sample size was determined for the primary outcome of self-reported follow-up visit to a GP at any time between baseline and three months. To conduct regression analysis, we estimated that at least 240 adults (120 per group) would be needed to detect a small to moderate difference (effect size 0.4) in follow-up behaviours between groups with a two-tailed α of 0.05 and a 1-β of 0.95. With very few previous studies of uptake of diabetes risk assessment tools, we assumed a 30% attrition between baseline and three-month follow-up, based on previous studies by the authors [43].

Randomisation (*Sequence generation*, *allocation concealment mechanism*, *and implementation)* Website auto-allocation was used to generate the random order for allocation of participants After screening, all eligible participants with a high-AUSDRISK score (≥12 or points) were allocated to a holding group where an alphanumerical participant identification (ID) was generated prior to randomisation to either the: (i) intervention group: video-based story AND text-based message (VS+TM) or (ii) control group: text-based message only (TM).

Website auto-allocation meant that allocations were concealed from the researchers. Block randomisation (blocks of six) with equal allocation of participants to either of the two groups, was used to ensure balance in the sample size of each group as they were recruited over the six-month recruitment period.

### Statistical methods

SAS Software v9.4, SAS institute Inc., Cary, NC, USA was used for data analysis. Following Intention-to-Treat principles, data from all participants (N = 477) were used in the analysis. Descriptive statistics were presented as frequencies and percentages by treatment groups for categorical variables, and as means and standard deviations for continuous variables. Differences in visiting GP and health professional were tested using chi-squared tests. Generalized linear mixed models (GLMM) with gamma distribution and log link were run separately for each continuous outcome (i.e., self-reported physical activity minutes and BMI) [44]. Robust standard errors were calculated using empirical estimator. Mean ratios and 95% CI were reported. GLMM with binomial distribution and logit link were also run separately for each dichotomous outcome (i.e., meeting the guidelines for physical activity, fruit, vegetables, and fruit/vegetables). Odds ratios and 95% CI were reported. Each model included fixed effects for group, time, and an interaction term between group and time. Subject was considered as random effect. Adjustment for multiple comparisons between subgroups were made using the simulation option available in PROC GLIMMIX. Multiple imputation (MI) using chained equations with the assumption of missing at random were used to impute missing values [45]. Baseline values and demographic characteristics were used to inform imputation models. Sensitivity analysis (See S2 File) was conducted on 50 imputed datasets using the same statistical models as being used for original data. All p-values were two-tailed, and the significance level was set at 0.05.

## Results

**Fig 1:**
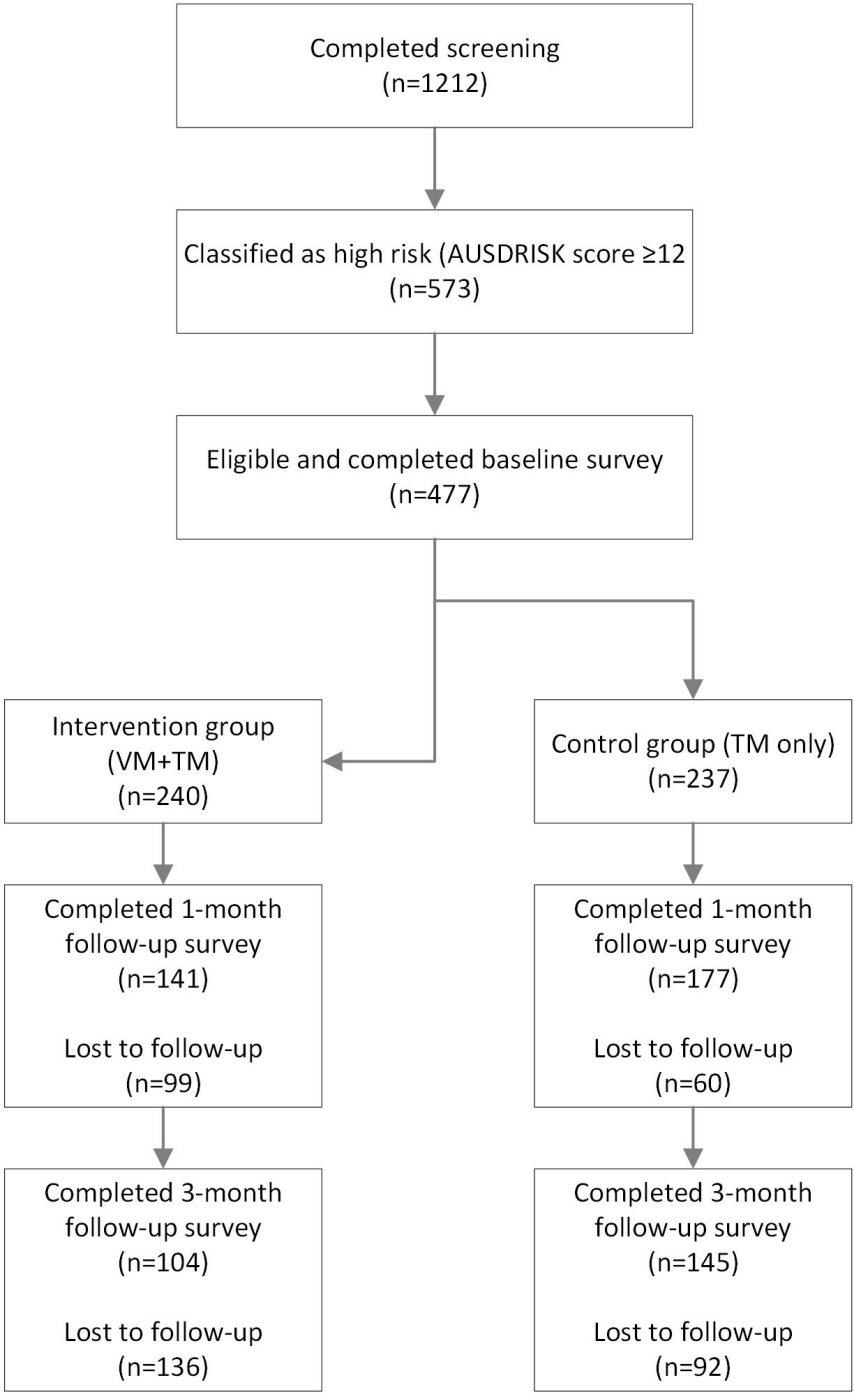
Flowchart of participation.

Fig 1 shows the number of participants at each assessment point. A total of 1212 adults (81% female) completed the initial screening. Of these, 573 were classified with a high-risk score (12 or more points) after completion of the AUSDRISK and 477 agreed to participate and completed the baseline survey (240 randomised to intervention group (VS+TM); 237 randomised to control group (TM only). Of these participants, a total of 318 completed the one-month follow-up survey (VS+TM (n = 141); TM only (n = 177)) and 249 completed the three-month follow-up survey (VS+TM (n = 104); TM only (n = 145)). More participants in the control group (TM only) (n = 140) compared to the intervention group (VS+TM) (n = 94), completed all surveys in the study.

The characteristics of participants at baseline are shown in Table 1. The average age of participants was 52.3 (SD ±9.6) years, and most were female (78%), partnered (married/informal relationship) (73%) and had attained a tertiary education (68%). Half of the participants were classified as middle-income earners (50.4%) and one third as high-income earners (34.7%). There was almost equal representation from different living locations (major city (31.2%), Inner regional towns (38.6%) and Outer regional/remote towns (30.2%). The mean eHealth literacy score was 32.9 (SD±4.3). Just under half of all participants were classified as insufficiently active (46.8%), the majority did not meet recommendations for daily fruit (84.1%) or vegetable intakes (90.8%) and were classified as overweight (Body Mass Index (BMI) 25–30 kg/m^2^) (32.1%) or obese (BMI>30kg/m^2^) (57.6%). The average diabetes risk score was 16 (the highest score was 29). Just over half of participants had a risk score between 12 and 15 (56.0%), a further 27.2% had a risk score between 16 and 19 and the remainder (16.8%), had a risk score greater than 20.

**Table 1:** Baseline characteristics of participants, by group (N = 477).

	Total	Control group^a^		Intervention group^b^	
Characteristic	% or Mean (SD)	n	% or Mean (SD)	n	% or Mean (SD)
Age (mean ± SD) years	52.3 (9.6)	237	52.5 (9.9)	240	52.2 (9.3)
Gender					
Male	23.5	59	24.9	53	22.1
Female	76.5	178	75.1	187	77.9
Highest level of education (n = 474)					
High school/below	17.7	39	16.6	45	18.8
Trade/technical studies	13.9	33	14.0	33	13.8
Tertiary	68.4	163	69.4	161	67.4
Employment					
Unemployed/other	15.0	46	19.4	34	14.2
Part-time/Casual	21.0	50	21.1	48	20.0
Full time	64.0	141	59.5	158	65.8
Household income					
Low < $1,000/week	14.9	34	16.6	27	13.2
Middle $1,000 - <$2,500/week	50.4	67	32.7	78	38.2
High ≥ $2,500/week	34.7	104	50.7	99	48.5
Marital status					
Partnered	73.1	176	74.9	169	71.3
Un-partnered	26.9	59	25.1	68	28.7
Living location					
Major city	31.2	82	34.6	67	27.9
Inner regional	38.6	91	38.4	93	38.8
Outer regional/remote	30.2	64	27.0	80	33.3
eHealth literacy (mean ± SD)	32.9 (4.3)	237	32.9 (4.2)	240	33.1 (4.6)
Meeting health behaviour guidelines					
Sufficiently active	53.2	71	51.5	80	55.0
Daily fruit serves (2/day)	15.9	40	16.9	36	15.0
Daily vegetable serves (5/day)	9.2	23	9.7	21	8.8
Body Mass Index (BMI) (mean ± SD)	32.3 (6.3)	237	32.6 (6.5)	240	31.9 (6.2)
Healthy weight^c^	29.4	27	10.9	23	9.2
Overweight^d^	33.7	70	28.3	90	35.9
Obese^e^	36.9	150	60.7	138	55.0
Diabetes risk score (mean ± SD)	16.0 (3.6)	237	15.9 (3.6)	240	16.1 (3.5)
Scores 12–15	56.0	137	57.8	130	54.2
Scores 16–19	27.2	64	27.0	66	27.5
Scores 20+	16.8	36	15.2	44	18.3

a Control group = TM only—text-based message only.

b Intervention group = VS+TM—video story + text-based message.

c Healthy weight– 18.5–24.99 kg/m^2^.

d Overweight = 25–29.99kg/m^2^.

e Obese = ≥30 kg/m^2^.

For both groups combined, a total of 214/477 participants (44.9%) acted by visiting a GP and/or other health professional during the 3-month period. A total of 175/477 participants (36.7%) reported visiting their GP and 148/477 participants (31.0%) reported visiting other health professionals during the 3-months of follow-up. Just over half (50.9%) of the 214 participants who took any action, visited both their GP and another health professional. Table 2 outlines follow-up visitations with a GP and other health professionals at one- and three-months, by group. The number of participants who did not visit either a GP or health professional was substantially higher in the VS+TM group (60.0%) compared to the TM only group (50.2%). However, chi-squared tests showed no significant differences (*p* = 0.18) between the two groups at 1 and 3 months, in the proportion of those who visited their GP and/or a health professional, GP only, other health professionals only, and those who did not visit either.

**Table 2:** One and three-month follow-up visitations with General Practitioner (GP) and other health professionals, by group (N = 477).

	TM only group (n = 237)				VS+TM group (n = 240)			
	Action taken after 1 month		Action taken after 1 and 3 months combined		Action taken after 1 month		Action taken after 1 and 3 months combined	
***Follow-up visitation***	n	%	n	%	n	%	n	%
GP and other health professional	37	15.6	59	24.9	33	13.8	50	20.8
GP only	34	14.3	36	15.2	27	11.3	30	12.5
Other health professional only	20	8.4	23	9.7	20	8.3	16	6.7
Did not visit either	146	61.6	119	50.2	160	66.7	144	60.0

a Control group = TM only—text-based message only.

b Intervention group = VS+TM—video story + text-based message.

Note: Chi-square tests showed no significant differences (*p* = 0.18) between the TM only group and the VS+TM groups at 1 and 3 months, in the proportion of those who visited their GP and/or a health professional, GP only, other health professionals only, and those who did not visit either.

Table 3 outlines effectiveness of the intervention on improving body weight status (BMI) and health outcomes (total weekly physical activity minutes, meeting recommendations for weekly physical activity and daily fruit and vegetable intakes). For all outcomes, there was no interaction effect (p-values>0.05) that is, the messaging was not more effective for the intervention group (VS+TM) compared to the control group (TM only). However, there were time effects for all outcomes (p-values<0.0001). That is, all outcomes in both the text-based message and the video-story and text-based message groups were improved at the end of the three months following receipt of their risk score.

**Table 3:** Effects of intervention on Body Mass Index, physical activity and fruit and vegetable intakes, by group (n = 477).

	TM only group (n = 237)			VS+TM group (n = 240)			Time effect *p*	Group effect *p*	Interaction *p*
	Baseline	1 month	3 months	Baseline	1 month	3 months			
Body Mass Index (kg/m^2^)	32.63 (6.51)	n/a	32.00 (6.31)	31.91 (6.16)	n/a	31.55 (5.33)	<0.0001	0.1782	0.3700
Meeting guidelines for physical activity (PA)									
No	48.52	34.46	26.90	45.0	31.91	30.77	<0.0001	0.9771	0.5701
Yes	51.48	65.54	73.10	55.0	68.09	69.23			
Total PA minutes	272.28 (276.76)	358.93 (312.41)	360.76 (287.98)	272.13 (257.80)	329.69 (266.60)	358.17 (290.27)	<0.0001	0.9152	0.6826
Meeting guidelines for daily fruit intake									
No	83.12	68.36	64.83	85.0	64.54	66.35	<0.0001	0.9709	0.6327
Yes	16.88	31.64	35.17	15.0	35.46	33.65			
Meeting guidelines for vegetable daily intake									
No	90.30	75.71	74.48	91.25	83.69	76.92	<0.0001	0.3017	0.5353
Yes	9.70	24.29	25.52	8.75	16.31	23.08			
Meeting guidelines for daily fruit and vegetable intakes									
No	78.90	55.93	54.48	79.17	59.57	54.81	<0.0001	0.7917	0.8474
Yes	21.10	44.07	45.52	20.83	40.43	45.19			

NOTE: The columns headed ‘Time effect’, ‘Group effect’ and ‘Interaction’ provide the p-values for differences over time, differences between groups and group x time interaction effects.

Between baseline and three months, there was a net decrease in BMI of 0.63 kg/m^2^ and 0.36 kg/m^2^ in the TM only and VS+TM groups, respectively, a net increase in total weekly physical activity of 88.48 minutes (12.64 minutes/day) in the control TM only group and 86.04 minutes (12.29 minutes/day) in the VS+TM group, and increase in the proportion of participants meeting recommendations for physical activity in the control TM only group from 51.5% to 73.0% and 55.0% to 69.1% in the VS+TM group. The proportion of participants meeting recommendations for fruit and vegetable intake also increased between baseline and three months: in the control TM only group from 16.9% to 35.2% (fruit) and 9.7% to 25.5% (vegetables) and the VS+TM group from 15.0% to 33.6% (fruit) and 8.7% to 23.1% (vegetables).

Effect of the intervention over time on BMI, physical activity and fruit and vegetable intakes are presented in Table 4. Between baseline and three-months, there was a significant increase in total weekly physical activity time in both the TM only (45%, p<0.001) and VS+TM groups (33%, p<0.001) and for BMI, a decrease of 1% (p<0.001) in the control TM only group and 2% (p<0.001) in the VS+TM group. Similarly, participants were more likely to meet the physical activity guidelines and the fruit/vegetable guideline at one month and three months for both groups, with ORs ranging between 1.88 to 3.47. Results from sensitivity analysis were similar to those from the main analysis (See S2 File).

**Table 4:** Time effect for Body Mass Index, physical activity and fruit and vegetable intakes, by group.

	Control				Intervention			
	1 month vs. baseline		3 months vs baseline		1 month vs. baseline		3 months vs baseline	
	Estimate	p-value	Estimate	p-value	Estimate	p-value	Estimate	p-value
Total PA time/week*	1.36 (1.21, 1.52)	<0.001	1.45 (1.28, 1.64)	<0.001	1.29 (1.12, 1.48)	<0.001	1.33 (1.14, 1.56)	<0.001
BMI*	n/a		0.99 (0.98, 0.99)	0.001	n/a		0.98 (0.97, 0.99)	<0.001
Meeting PA guideline (yes vs. no)^+^	2.06 (1.31, 3.23)	0.001	3.08 (1.86, 5.11)	<0.001	1.88 (1.16, 3.07)	0.010	2.06 (1.19, 3.59)	0.012
Meeting fruit guideline (yes vs. no)^+^	2.43 (1.47, 4.02)	<0.001	2.79 (1.67, 4.68)	<0.001	3.38 (1.97, 5.81)	<0.001	2.91 (1.6, 5.29)	<0.001
Meeting vegetable guideline (yes vs. no)^+^	3.25 (1.79, 5.88)	<0.001	3.44 (1.84, 6.44)	<0.001	2.03 (1.03, 3.97)	0.039	3.18 (1.58, 6.41)	0.001
Meeting fruit/vegetable guideline (yes vs. no)^+^	3.37 (2.10, 5.40)	<0.001	3.47 (2.11, 5.69)	<0.001	2.81 (1.69, 4.67)	<0.001	3.46 (1.99, 6.03)	<0.001

Analyses of data from all participants followed Intention-to-Treat (ITT);

*Means Ratios presented for total PA time/week and BMI; ^+^Odds Ratios presented for meeting PA, fruit and vegetable guidelines.

## Discussion

The aim of this study was to examine the follow-up behaviours of individuals classified at high risk of type 2 diabetes and differences in follow-up behaviours after receiving either a text-based only message or video-based story combined with a text-based message following completion of the online AUSDRISK. The primary hypothesis was not confirmed: the intervention effect (follow-up with a medical professional, changes in health status (BMI) and changes in health behaviours (dietary intake, physical activity) were not significantly higher in the video-based story and text-message (VS+TM) group compared to the text-message only (TM) group. Although no significant differences were found between the control and interventions groups, the results of this study suggest a strong positive effect of the diabetes risk assessment and messaging on BMI and related health behaviours (physical activity and nutrition intakes) in both study groups. In line with previous research of diabetes risk assessment [9, 10] our findings support the role of personalised risk calculators (irrespective of delivery mode) in motivating individuals to take actions to reduce their risk [11].

When considered at a population level and based on the known links between lifestyle behaviours and type 2 diabetes incidence [2], our findings highlight the utility of the AUSDRISK in motivating some individuals to take action to reduce their risk [11]. Our findings do suggest that the AUSDRISK has the potential to reduce the burden of diabetes when used broadly across the population. As very little is known about the reach and extent of use of the AUSDRISK in Australia, further research and reporting of use in the general population is needed to fully appreciate this potential.

In our study, the absence of significant differences in follow-up behaviours of individuals who received a personalised video-based risk message in combination with a text-based message versus a non-personalised text-based risk message (and tendency for more of those in the non-personalised text-based message to take further action), contrasts with previous studies that have found audio-visual format effective for communicating health information to those with low health literacy [23, 46]. This disagreement may relate to the generally high levels of healthy literacy and education in our study sample. Based on previous studies [47, 48] irrespective of the type of message received, individuals with higher health literacy may have greater capacity to understand their risk and the messages provided to them and have more knowledge about the actions they need to take to reduce their risk. This suggests that in our study participant understanding of their risk and the message provided to them, was not a barrier to taking further action. Further investigation of characteristic differences between those who did and did not take action is warranted.

Our lack of significant differences between the control and intervention group (who received a video-based message) also aligns with a recent study that included personally tailored videos for physical activity [49] but contrasts with earlier studies that have found that personalisation of risk assessments [11] and/or video-based messages more effective for health communication and promotion than text-based only messages for obesity prevention [21, 25], dietary behaviour change [28] and smoking cessation [26]. With only 13 different scenarios and five main risk factors for type 2 diabetes (age, gender, family history/gestational diabetes, weight status and physical activity and nutrition behaviours) included in the video stories, our findings may also reflect a lack of sophistication of the video-tailoring that occurred in the DORA study. Including more specific evidence-based behaviour change techniques (e.g., goal setting) at the time of messaging, may be necessary to increase an individual’s self-efficacy [27]. Providing more specific information such as the meaningful rationale for why behaviour change is important, explanation and/or more information about what to do next (after receipt of message and risk score) [13] and acknowledgement of the difficulties faced in changing behaviours, may be needed to enhance impact of the personalised message [50]. In addition, based on an earlier study of risk assessments for coronary heart disease [19], impact of the AUSDRISK may be further enhanced if the risk information and messaging is repeated overtime rather than as a one-off message—as occurred in our study. Further research is needed to understand if providing feedback with higher levels of personalisation and/or repetition of messaging overtime can increase effectiveness of diabetes risk assessment tools.

The overall proportion of people with a high-risk score who followed-up with their GP was similar to an Australian report [18] of adults with moderate or high-risk scores. Under-reporting of follow-up actions may have occurred in our study as the three-month follow-up period may not have provided enough time for participants to arrange and secure appointment times with their GP. This is supported by reasons reported by participants in our study who did not follow-up with their GP including: being too busy, still waiting for their next doctor’s appointment, or unable to get to see a doctor. Based on these comments from participants and previous research [20], a longer follow-up period of at least 6 to 12 months may be necessary to allow more time for an individual to take action and to better understand longer term effectiveness of the risk messaging. Future studies should include investigation of barriers to action to better understand how messages can be tailored.

### Strengths and limitations

Key strengths of this study include its design in the context of a real-world web-based online risk assessment for type 2 diabetes, and an almost equal representation of participants from metropolitan, regional, and rural locations across Australia. The authors acknowledge several limitations. The use of self-report for all study outcomes may have been affected by recall bias and produced inaccuracies in estimates of associations between follow-up and other health behaviours [51]. A short, three-month follow-up period was chosen for this study to support examination of immediate changes in health behaviours and follow-up actions following receipt of the risk message. A longer period of follow-up may be necessary for understanding the true benefits of risk-messaging and sustainability of changes in health behaviours [52]. We also experienced high attrition from baseline to three-months (52%) and more than what was presumed at commencement of the study. The loss of participants is not uncommon however in this study, the high rate of attrition may suggest a mismatch between the intervention and participant needs and expectations [13] but may also be a natural and typical feature of online risk assessments and reflect how these tools are used and viewed in real-world settings. Additional limitations include not having a true control group (i.e., no message provided) and use of self-report measures for height and body weight. Physical activity and dietary behaviours were also self-reported, however the measures applied have shown to be valid and reliable [39–41]. We are also unable to discern if participants who reported follow-up actions at 1-month, also reported repeat visits with their GP or other health professionals at 3-months and this may have resulted in over-reporting of these actions. In this study, similar to other organisational websites which include the AUSDRISK, links to reputable information sources such as government health websites and online health programs that encourage healthy lifestyle behaviours, were provided to participants via the study website. Uptake of these resources could not be monitored hence, any influence of these resources on study outcomes is unknown. Due to the eligibility criteria included in this study, which was conducted wholly via online platforms, the findings of this study are not generalisable to all population groups with high risk of type 2 diabetes. In particular, those with low health literacy and/or who do not have access to a reliable internet connection and those unable to comfortably speak and read the English language.

## Conclusions

No previous studies have explored the use of tailored video-based storytelling for diabetes prevention nor investigated the impact of messages relating risk assessment scores, on follow-up behaviours of those who complete an online diabetes risk assessment. As a first study to investigate the follow-up behaviours of individuals completing an online version of the AUSDRISK our results validate the positive role of these types of self-assessment tools in promoting lifestyle behaviour change in high-risk individuals and promoting early detection of glucose intolerance or pre-diabetes. Our study also provides evidence that the AUSDRISK can be effective in promoting short-term positive changes in nutrition and physical activity behaviours which can contribute to an overall reduction in the development of type 2 diabetes.

## Supporting information

S1 File Text-based message and letter. (PDF)

S2 File Sensitivity analysis. (PDF)

S3 File Study protocol. (PDF)

S1 Checklist CONSORT 2010 checklist of information to include when reporting a randomised trial*. (PDF)

S1 Appendix Data repository. (XLSX)
