# Supplementary material for: What is the effectiveness of a personalised video story after an online diabetes risk assessment? A Randomised Controlled Trial
Source: PLoS One. 2022 Mar 3;17(3):e0264749. doi: 10.1371/journal.pone.0264749 (PMC8893700; doi:10.1371/journal.pone.0264749)
Supplement: S1 File — (PDF) [file pone.0264749.s001.pdf]

Supporting information 1

Text-based message and letter

Thank you for completing the Baseline survey and thank you for participating in the DORA Project.

The total points from your risk assessment have been calculated and your score shows that you are currently at HIGH or VERY HIGH risk of type 2 diabetes*.

*It is important to acknowledge that this is a risk rating and is NOT a diagnosis of type 2 diabetes.

So, what does this risk mean?

A high-risk rating means that genetic factors and current lifestyle factors are currently placing you at a higher risk of developing type 2 diabetes within the next 5 years.

- At medium risk - approximately one person in every 30 classified as medium risk will develop diabetes.
- At high risk - approximately one person in every 14 classified as high risk will develop diabetes.
- At very high risk - approximately one person in every 3 classified as very high risk will develop diabetes.
- Factors which increase your risk of type 2 Diabetes
- Genetic factors: such as age, gender, ethnicity, region of birth and family history are all genetic factors that contribute towards your risk of developing type 2 diabetes. These are the factors you cannot change, so it is important to concentrate on the lifestyle behaviours to reduce your risk.
- Lifestyle factors: such as high blood glucose, smoking, high blood pressure, fruit and vegetable intake, physical activity, weight and waist circumference influence your risk of developing type 2 diabetes.

Adopting healthy lifestyle behaviours, weight management, exercise and healthy eating make a significant impact on risk reduction now and into the future.

What you should do next….

Given that your risk assessment indicates that you are at an elevated risk of type 2 diabetes, the DORA Project recommends you make an appointment with your doctor to further discuss your risk. Your doctor will be in the best position to know your current health status and risk factors and recommend changes you may need to make to reduce your risk. We have attached a letter which you may take to your doctor which explains the DORA Project and the method used to arrive at the risk rating. Click on this link to download and print the DORA Letter to Doctor. The letter is also saved in the Library section of the DORA website.

As mentioned, it is important to acknowledge that this is a risk rating only based on your responses and is NOT a diagnosis of type 2 diabetes. A diagnosis can only be made by a medical professional following further testing.

Navigating health information on the internet can be daunting. For this reason, we highly recommend you access the following document with links to credible and reliable internet sources of information about diabetes and lifestyle factors. Links to health information

Information from these sites will help you understand your risk and changes which can be made to lower your risk. Use them in conjunction with recommendations made by your doctor. The website links document is also saved in the Library section of the DORA website.

To continue your involvement in the DORA Project, we request that you complete our brief Feedback Survey that follows on from this page.

This helps us to understand whether the information provided has been useful and encourages you to seek further information about your risk rating. If you are not able to complete the Feedback Survey now, it will be available on your DORA home page.

A summary of the requirements of the DORA Project and the Participant information sheet can be found in the Library section.

If you have any queries, please contact the DORA Project office …
