# Supplementary material for: What is the effectiveness of a personalised video story after an online diabetes risk assessment? A Randomised Controlled Trial
Source: PLoS One. 2022 Mar 3;17(3):e0264749. doi: 10.1371/journal.pone.0264749 (PMC8893700; doi:10.1371/journal.pone.0264749)
Supplement: S2 File — (PDF) [file pone.0264749.s002.pdf]

Supplementary information 2

Sensitivity analysis

	1 month vs. baseline	3 months vs baseline
Total PA time/week		
Within control	1.34 (1.19, 1.52)***	1.43 (1.25, 1.65)***
Within intervention	1.23 (1.06, 1.42)**	1.27 (1.07, 1.51)**
Between intervention - control	0.91 (0.76, 1.11)	0.89 (0.71, 1.09)
BMI+		
Meeting PA guideline (yes vs. no)		
Within control	1.92 (1.26, 2.94)**	2.86 (1.79, 4.57)***
Within intervention	1.82 (1.16, 2.83)**	1.77 (1.05, 2.97)*
Between intervention - control	0.95 (0.52, 1.75)	0.62 (0.31, 1.25)
Meeting fruit guideline (yes vs. no)		
Within control	2.39 (1.49, 3.82)***	2.80 (1.73, 4.48)***
Within intervention	3.49 (2.14, 5.64)***	3.03 (1.84, 5.05)***
Between intervention - control	1.45 (0.74, 2.86)	1.09 (0.55, 2.16)
Meeting vegetable guideline (yes vs. no)		
Within control	3.35 (1.92, 5.81)***	3.49 (1.95, 6.17)***
Within intervention	2.12 (1.15, 3.94)*	3.13 (1.67, 5.87)***
Between intervention - control	0.63 (0.28, 1.45)	0.90 (0.39, 2.10)

***p<0.001, **p<0.01, *p<0.05

+Not applicable due to between-imputation variance is nearly zero
