# Supplementary material for: What is the effectiveness of a personalised video story after an online diabetes risk assessment? A Randomised Controlled Trial
Source: PLoS One. 2022 Mar 3;17(3):e0264749. doi: 10.1371/journal.pone.0264749 (PMC8893700; doi:10.1371/journal.pone.0264749)
Supplement: S3 File — (PDF) [file pone.0264749.s003.pdf]

HUMAN RESEARCH ETHICS COMMITTEE

Negligible and Low Risk Review Process and Application Form

Please write in BLOCK letters using a black pen or TYPE.

Signatures can be inserted electronically (soft copy) or sign a hard copy (paper).

The National Health and Medical Research Council (NHMRC) 'National Statement on Ethical Conduct in Human Research', 2007 (the National Statement') recognises that human research involves a wide range of activities that have variable risks and potential benefits. The National Statement establishes different levels of ethical review, based on the degree of risk involved.

There are three levels of risk:

- Harm;
- Discomfort; and
- Inconvenience.

Researchers and HRECs are required to determine the existence, likelihood and severity of these risks based on the research methodology and design, participant population and research activity. The National Statement, sections 2.1.6-2.1.7 holds that:

> *2.1.6 Research is 'Low Risk' where the only foreseeable risk is one of discomfort. Where the risk, even if unlikely, is more serious than discomfort, the research is not low risk.*
>
> *2.1.7 Research is 'negligible risk' where there is no foreseeable risk of harm or discomfort; and any foreseeable risk is not more than inconvenience. Where the risk, even if unlikely, is more than inconvenience, the research is not negligible risk'.*

Research that involves the risk of harm or the likelihood of harm must be reviewed by a fully constituted HREC. For research involving only the risk of inconvenience, or discomfort (i.e., low or negligible risk), Institutions may establish an alternative ethical review process. CQUniversity has resolved to proceed with such an alternative process.

It should be noted that research involving certain groups, methodologies or procedures, regardless of the level of risk, must be reviewed by a full HREC (Clause 5.1.6 of the National Statement).

There are a range of resources available to researchers on the Human Research Ethics Committee webpage (http://www.cqu.edu.au/research/current-research-staff/committees-and-ethics/human-research-ethics-committee), including sample information sheets, consent forms and an example of a completed Low Risk Application form. Researchers are encouraged to complete the checklist first and consult with the Ethics Officer to gain an assessment of whether the project satisfies the criteria for alternative review. Time constraint is NOT an acceptable reason for seeking review through this process.

Process Flowchart

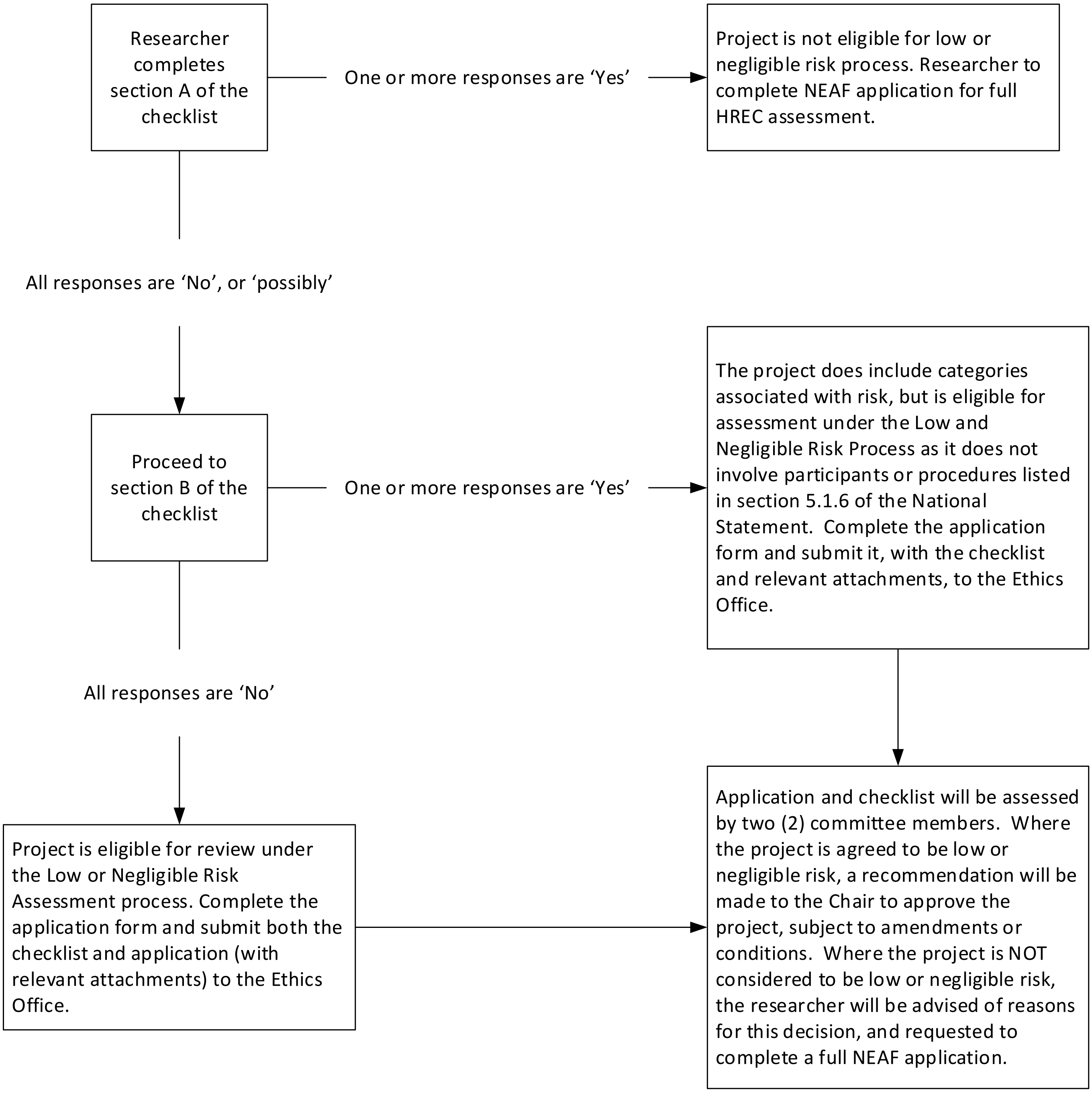

LOW OR NEGLIGIBLE RISK ASSESSMENT PROCESS CHECKLIST

SECTION A

Please indicate whether your project involves any of the following:

YES	NO	
[ ]	[X]	Some form of deception is involved
[ ]	[X]	The project involves interventions and/or therapies, including clinical and non-clinical trials and innovations, human genetics or human stem cells

Please indicate whether your project is actively seeking to recruit participants meeting the criteria below. Note – if it is possible that participants may meet one or more of these criteria as a result of being part of the general population, you should tick the 'possibly' box.

YES	Possibly	NO	
[ ]	[ ]	[X]	Participants are aged less than 18 years
[ ]	[ ]	[X]	Participants are cognitively or emotionally impaired, or are highly dependent on medical care
[ ]	[X]	[ ]	Participants belong to the Aboriginal or Torres Strait Islander People
[ ]	[X]	[ ]	Female participants who are pregnant and/or the human foetus
[ ]	[ ]	[X]	Participants who may be involved in illegal activities, where the research is intended to study or expose illegal activity

IF you have answered YES to any of the above, your project CANNOT be considered under the Low or Negligible Risk Assessment Process, and you must lodge a NEAF application to the Human Research Ethics Committee.

If you have answered either 'NO' or 'Possibly' to all of the above, please proceed to Section B.

SECTION B

Are any of the following topics covered in part or in whole?

YES	NO	
[ ]	[X]	Research about parenting issues
[ ]	[X]	Research investigating sensitive personal or cultural issues
[ ]	[X]	Explorations of grief, death or serious/traumatic loss
[ ]	[X]	Mental disorders, e.g., depression, mood states, anxiety
[ ]	[X]	Gambling
[ ]	[X]	Eating disorders
[ ]	[X]	Illicit drug use/Substance abuse (prescription or over the counter)
[ ]	[X]	Self report of criminal behaviour
[ ]	[X]	Any psychological disorder
[ ]	[X]	Suicide risks/Anger management
[ ]	[X]	Gender identity/Sexuality
[ ]	[X]	Race or ethnic identity
[X]	[ ]	Any disease or health problem
[ ]	[X]	Fertility/Termination of pregnancy

If you have answered 'yes' to any of the topics listed above: Will this research be conducted online or via other anonymous recruitment?

**NO**	☐	**your project CANNOT be considered under the Low or Negligible Risk Assessment Process, and you must lodge a NEAF application to the Human Research Ethics Committee.**
**YES**	☒	**your project may meet the criteria for Low Risk. To enable the committee to assess eligibility, please provide one reference which validates the use of your chosen research instrument:**

Key processes and outcomes of this study relate to online access of health information which is already provided to the Australian population, therefore online data collection methods are appropriate. Example: Diabetes Queensland – Assess your risk of developing type 2 diabetes http://www.diabetesqld.org.au/healthy-living/who-is-at-risk/assess-your-risk.aspx

SECTION C

Are any of the following procedures to be employed?

YES	☐	NO	☒	Use of personal data obtained from Commonwealth or State Government Department/Agency with participant consent
YES	☐	NO	☒	Deception of participants or concealing the purposes of the research
YES	☐	NO	☒	Covert observation (or minimal disclosure)
YES	☐	NO	☒	Audio or visual recording without consent
YES	☐	NO	☒	Recruitment of a third party or agency (asking participants to provide information about another person)
YES	☐	NO	☒	Withholding from one group specific treatments or methods of learning from which they may 'benefit' (e.g., in medicine or teaching)
YES	☐	NO	☒	Psychological interventions or treatments
YES	☐	NO	☒	Application of physical stimulus/Invasive physical procedures/Infliction of pain
YES	☐	NO	☒	Administration of drugs/Administration of other substances or devices
YES	☐	NO	☒	Exposure to ionising radiation
YES	☐	NO	☒	Tissue sampling or blood for pathological or genetic testing
YES	☐	NO	☒	Collecting body fluid (e.g., saliva)
YES	☐	NO	☒	Use of medical records where participants can be identified or linked

Other Risks:

YES	☐	NO	☒	Are there risks to the researcher? (e.g., research conducted in unsafe environments or trouble spots)
YES	☐	NO	☒	Are there risks to non participants in the research, such as participant's family members and social community? (e.g., effects of biography on family and friends or infectious disease risk to the community)
YES	☐	NO	☒	Is the research being conducted overseas ?

Select the categories of people that are targeted or likely to be targeted as participants in this research project

YES	☐	NO	☒	Suffers from a psychiatric or psychological disorder
YES	☒	NO	☐	Suffering a physical disability or medical condition
YES	☐	NO	☒	Children and/or young people without parental or guardian consent
YES	☐	NO	☒	Resident of a custodial institution
YES	☐	NO	☒	Unable to give freely an informed consent because of difficulties in understanding information provided (e.g., language difficulties, NESB)
YES	☐	NO	☒	Members of a socially identifiable group with special cultural or religious beliefs or political vulnerabilities
YES	☒	NO	☐	Participants are identifiable or re-identifiable, (eg oral history projects), or data will be kept in re-identifiable form, to enable longitudinal comparisons to be made (not identifiable in final report) . If yes, please ensure that you address this in question 7.1
YES	☐	NO	☒	Participants are potentially identifiable in final report when specific consent for release has not been given
YES	☐	NO	☒	Those in a dependent relationship with the researchers (e.g., lecturer/student, doctor/patient, teacher/pupil and professional/client)

If 'NO' has been selected for all questions in sections A B and C, the project IS eligible for review under the Low or Negligible Risk Assessment process. Please complete the Application Form which follows and submit this entire document, together with a copy of the Information Sheet, Consent form and research instrument to the Ethics Office.

If you have selected 'YES" to any of the items in Section B or C, the project MAY STILL BE ELIGIBLE for assessment under the Low or Negligible Risk Assessment Process. Please complete the Application Form which follows and submit this entire document, together with a copy of the Information Sheet, Consent form and research instrument to the Ethics Office.

LOW OR NEGLIGIBLE RISK ASSESSMENT PROCESS
APPLICATION FORM

This form is to be completed for research involving no more than low or negligible risk, as identified by completion of the checklist on the preceding pages.

If you are a staff member seeking to survey students enrolled in courses that you have responsibility for, please ensure that in section 2.7 of the form you acknowledge that there is a power differential between the researcher and participant, and address how you will minimise the potential for students to feel obligated to participate.

Please ensure that:

- ❖ You have attached the completed checklist.
- ❖ All signatures have been obtained.
- ❖ You have included copies of Information Sheets, Consent Forms, Research Instruments (survey, interview questions, etc.) and approvals from participating organisations (as appropriate).
- ❖ You do not commence data collection until written approval has been received from the Chair of the Human Research Ethics Committee.

Return to:

Ethics Office
Research Division (Bldg 32 Rm 2.60)
CQUniversity Australia
Rockhampton Queensland 4701

Phone: 07 4923 2603
Fax: 07 4923 2600
Email: ethics@cqu.edu.au

PART 1 RESEARCHERS

Principal Researcher (or principal supervisor if this is a student project):

Title	Dr.
Name	Susan L Williams
Telephone	07 4923 2213
Facsimile	
Email	s.p.williams@cqu.edu.au
Current Qualifications	PhD, RPHNutr
Research experience	Dr. Williams' research encompasses the areas of nutrition, health and obesity related behaviours. Dr. Williams has experience in conducting online surveys and population based studies. Dr. Williams has 20 years of clinical nursing experience and 12 years of experience in academic research and teaching.
If this relates to a student project, specify program	N/A

Other Researchers (if this is a student project, the student and any other supervisors need to be included here):

Title	Dr.	Title	Dr.
Name	Kate Ames	Name	Celeste Lawson
Telephone	07 3295 1148	Telephone	07 4923 2558
Facsimile		Facsimile	
Email	k.ames@cqu.edu.au	Email	c.lawson@cqu.edu.au
Qualifications	PhD	Qualifications	PhD
Research experience	Dr. Ames is an experienced qualitative researcher in cultural sociology, communication, and education with specific experience in focus group facilitation, interviews, and discourse analysis methods.	Research experience	Dr. Lawson is an active researcher with publications in international and national journals. Experience in social media research, policy analysis, interviews and systematic reviews at PhD and post-Doctoral leve

Role (student, supervisor, co-researcher)	Co-researcher	Role (student, supervisor, co-researcher)	Co-researcher

Title	Dr	Title	Professor
Name	Sonia Saluja	Name	Corneel Vandelanotte
Telephone	07 4930 6434	Telephone	07 4923 2183
Facsimile		Facsimile	
Email	s.saluja@cqu.edu.au	Email	c.vandelanotte@cqu.edu.au
Qualifications	MD	Qualifications	PhD
Research experience	Dr. Saluja is a medical academic with research interests in chronic disease, health literacy and medical education. Dr Saluja has 18 years experience in medical education and research.	Research experience	Prof Vandelanotte's expertise is in website- and app-delivered, as well as computer-tailored interventions for increasing physical activity at the population level. He has excellent experience and expertise with developing, implementing and evaluating affordable and effective health behaviour change interventions using innovative technologies to reach large populations.
Role (supervisor, co-researcher)	Co-researcher	Role (supervisor, co-researcher)	Supervisor/co-researcher

Authorisation from external organisations (if research is not conducted at CQUniversity locations)

Title	
Name	
Telephone	
Facsimile	
Email	
Position in organisation	

PART 2 PROJECT DETAILS

2.1 Project Title:

Online diabetes risk assessment

2.2 Layperson Description:

Briefly outline in simple terms the project's aim(s), justification, participant group(s), method and possible outcomes

Aim: to develop and examine usability and acceptability of an intervention that provides tailored video-based stories in combination with text-based feedback, to facilitate follow-up action and behaviour change in individuals who receive an intermediate or high risk score following completion of the Australian Type 2 Diabetes Risk Assessment Tool (AUSDRISK). **Primary research question**: Does the addition of video-based storytelling to text-based messages received after completion of the AUSDRISK, result in greater General Practitioner (GP) 'hit-rates' (i.e follow up with GP).

Justification: Type 2 diabetes mellitus (T2DM) is considered the epidemic of the 21st century and there are large numbers of people with silent, undiagnosed T2DM which may be damaging their bodies. An estimated 2 million Australians are at high risk of developing T2DM and are unaware of their risk and up to 58% of cases may be prevented if they are detected early. The AUSDRISK is a validated tool that was developed to assist in predicting five year risk of diabetes. Responses to the questions relating to known risk factors for T2DM permit calculation of an individual's risk of developing T2DM as either: low; intermediate; or high.

The AUSDRISK is currently available as an interactive online tool via a variety of health websites (e.g., Diabetes Queensland, Diabetes Australia) and once completed and individual receives their risk score and a static text-based message. According to data collected by Diabetes Queensland, only 36% of those receiving a

moderate/intermediate or high risk score from the AUSDRISK online, actually take action in following up with their General Practitioner (GP) to discuss their risk. This 'hit rate' is a limiting factor in the goal to reduce the incidence of T2DM.

By telling a good story that combines factual information with a human interest perspective we aim to make the health message received after completing the AUSDRISK more memorable and emotive. We will develop a series of digital stories that provide brief visual narratives that synthesise images, video, audio and text, to create compelling accounts of personal experiences that will be used to increase awareness and begin conversations around T2DM. These video-based stories will be delivered electronically to individuals who obtain an intermediate or high risk score following completion of the online AUSDRISK. Any individuals who receive a low risk score will be thanked for their participation and advised that they are ineligible to participate further in the study.

Study participants: will include Australian adults aged 35 years and older, who have no known diagnosis with T2DM but are considered at intermediate or high risk of developing T2DM (as determined by the AUSDRISK).

Methods: Participants will be initially recruited via online systems and all adults who are interested in participating in the study will be directed to the study website to complete a baseline survey and the interactive online AUSDRISK. Following completion of the AUSDRISK participants will receive either a video story (VS) + text message (TM) OR a text message (TM) only, that provides their diabetes risk score and information 'about what to do next'. Each video message will be tailored to age, gender and risk factors included in the AUSDRISK and will portray a 'similar' individual that has been diagnosed with T2DM and their personal history and journey from diagnosis to treatment and related experiences.

Possible outcomes:

From this study, we anticipate:

- advancing the knowledge base of health communication processes in the prevention of chronic diseases such as type 2 diabetes, and how to bridge the gap between individuals with low and high health literacy;
- enhancing current processes and practices used in the early detection of type 2 diabetes and implementing the AUSDRISK;
- contributing greater community understanding of type 2 diabetes through increased awareness and initiation of conversations following viewing of video-based stories; and
- preparing and submitting academic publications of results to highly ranked scientific journals.

2.3 Data collection dates:

Start December 1st, 2017 **End** December 31st, 2018

2.4 Data Collection methods: (Please tick methods as appropriate)

- ☐ Interviews
- ☐ Focus Groups
- ☐ Hard Copy Survey
- ☒ Online survey
- ☐ Archival data (please provide detail of where data sourced from, and evidence of permission from the owner of the data)

Other data collection techniques (eg observation, manipulation of environmental factors) (please specify)

2.5 Research Methodology:

Outline the proposed method, including data collection techniques, tasks participants will be asked to complete, estimated time commitment required of them; and how data will be analysed. Give a justification of your proposed sample size, including details of statistical power of the sample where appropriate

Data collection techniques:

Study participants

For this study we will use online methods (Facebook sites, community websites and email systems) to recruit a sample of Australian adults aged 35 years or older (based on age at which T2DM risk increases) who have no known diagnosis with T2DM (N=240). This sample size is based on G*Power (3.1.9.2) calculations for multiple regression analyses (α =0.05, power 0.95, 15 predictors). Recruitment of 120 adults per group (intervention vs usual care) and allows for a 30% attrition from baseline to final follow up at one and three months.

To ensure recruitment of individuals with intermediate or high risk of T2DM (in alignment with scores for related items on the AUSDRISK), all potential participants will be 'screened' for their internet access and their living location in Australia (to ensure only adults who are residing in Australia are recruited to the study).

All eligible participants will be provided with a username and password and directed to the intervention website and asked to complete a baseline survey and the online diabetes risk assessment (AUSDRISK) – approximate time for completion 20-25 minutes.

Once participants have completed their baseline survey and AUSDRISK they will automatically receive either (i) usual care group: a text-based only message (as currently occurs with other online AUSDRISK platforms) or (ii)

intervention group: a text + video-based message. Allocation to the usual care or treatment groups will be randomly generated.

Follow-up surveys will occur at one and three months following completion of their baseline survey. Participants will be asked to provide their email address and phone number to permit follow-up contact to be made.

Baseline and follow-up surveys (one and three months) will include a range of questions as outlined below:
The baseline survey will include:

1. The AUSDRISK (13 items).
2. Socio-demographic questions (education, income, family structure, and living locality)(7 items)
3. Diagnosis with chronic health conditions (1 item).
4. Dietary intakes in relation to recommendations for each food group (including intakes of discretionary foods) in the Australian Dietary Guidelines (39 item Food Frequency Questionnaire)(5 minutes completion time),
5. Physical activity behaviours - the Active Australia Survey will be used as a valid and reliable measure of adult physical activity (12 items).
6. Diabetes Symptom Checklist (DSC-R) (9 items).
7. Intention to change [(5 items) (from a previous study of diabetes risk perception and intention to adopt healthy lifestyles)].
8. Health literacy [(8) items from eHEALS).
9. Perceived risk of developing diabetes (3 items).
10. Need for cognition [(18 items) from the short-form of the Need-For-Cognition scale].
11. Acceptability of health message (19 items).

Each of the follow-up surveys (at 1 and 3 months post baseline) will ask participants to report their:

1. Follow up actions to visit their GP following notification of an intermediate or high-risk score after completion of the online risk assessment.
2. Any new diagnosis with chronic health conditions (1 item).
3. Dietary intakes in relation to recommendations for each food group (including intakes of discretionary foods) in the Australian Dietary Guidelines (39 item Food Frequency Questionnaire)(5 minutes completion time),
4. Physical activity behaviours - the Active Australia Survey will be used as valid and reliable measure of adult physical activity (12 items).
5. Diabetes Symptom Checklist (DSC-R) (9 items).
6. Intention to change [(6 items) (from a previous study of diabetes risk perception and intention to adopt healthy lifestyles)].
7. Perceived risk (3 items).

Data analysis: Data analysis using IBM SPSS v22 will include t-tests, linear mixed models, and regression analysis (applying intention-to-treat to account for missing data) to examine intervention efficacy, usability and acceptability.
Primary outcomes are:

1. Participants follow up actions to visit their GP for further testing for type 2 diabetes, and
2. Changes in the participants: dietary behaviours, physical activity, self-efficacy, intention, risk perceptions, and any symptoms of diabetes.
3. Acceptability of the intervention (health messages).

2.6 Research Aims and Significance:

State the aims, research objectives, key research questions, and significance of the project. Where relevant, state the specific hypothesis to be tested. Also, please provide a brief description of the relevance of your proposed project to current research, a justification as to why your research should proceed and an explanation of any expected benefits to the community or its potential to contribute to existing knowledge.

Aims:

To examine usability and acceptability of an intervention that provides tailored video-based stories (VS) in combination with text-based feedback (TM), to facilitate follow-up action and behaviour change in individuals who receive an intermediate or high risk score following completion of the AUSDRISK.

Objectives:

- To develop an intervention that provides tailored video-based stories in combination with text-based feedback following completion of the AUSDRISK.
- To enhance current processes, practices and outcomes (i.e., 'hit rates') used in implementing the AUSDRISK
- To advance the knowledge base of health communication processes in the prevention of chronic diseases such as type 2 diabetes.

Research questions:

- Does the addition of video-based storytelling to text-based messages received after completion of the AUSDRISK result in greater 'hit-rates' for the AUSDRISK? (ie. follow up with General Practitioner (GP) to discuss risk)
- Does the addition of video-based storytelling to text-based messages received after completion of the AUSDRISK lead to an improvement in nutrition and physical activity behaviours?

- Does the addition of video-based storytelling to text-based messages received after completion of the AUSDRISK increase intentions to change their nutrition and/or physical activity behaviours?
- Does the addition of video-based storytelling to text-based messages received after completion of the AUSDRISK increase individual health literacy?
- Does the addition of video-based storytelling to text-based messages received after completion of the AUSDRISK increase levels of diabetes risk perception?
- Does the participants need for cognition influence acceptance of health messages and follow-up actions?

Significance of the project:

This study is based on the concept of ‘a picture speaks a thousand words’ and literature which has found that the blending of music, images and text in a video-based story can provide greater dissemination of health promotion information in a more dynamic way than text only, and be more appealing to the those with lower health literacy.

No previous studies have explored the use of tailored video-based storytelling for diabetes prevention.

Demonstrating proof-of-concept in this study will allow for augmentation of current implementation processes used for the AUSDRISK to improve ‘hit-rates’ and greater effectiveness of this online tool in the prevention of T2DM.

Hypotheses:

1. Measures of intervention acceptability (will be significantly higher in the VS+TM group when compared to the TM-only group.
2. Measures of intervention effect (contact with medical professional/testing for T2DM, changes in related health behaviours (dietary intake, physical activity), and conversations with friends and/or family regarding T2DM) will be significantly higher in the VS+TM group when compared to the TM-only group.
3. Positive intervention effect of VS+TM when compared to the TM-only group will be greater in those with low health literacy at baseline.

2.7 Risk:

Please outline the likelihood and severity of the risks to participants/others. Please ensure that you address all forms of risk (harm, discomfort and inconvenience). Refer to section 2.1 of the National Statement. If you are a staff member seeking to survey students enrolled in courses that you have responsibility for, please ensure that you acknowledge that there is a power differential between the researcher and participant, and address how you will minimise the potential for students to feel obligated to participate.

1. Study participants are **at risk of being inconvenienced by the time burden** associated with their participation in the study (ie. they are will be asked to commit time to completing a total of three (3) surveys at baseline (approximate completion time 20-25 minutes), and one and three month follow ups (approximate completion time 20 minutes). All surveys will be tested on small groups prior to implementation to ensure this time expected for completion is correct.
2. Study participants are **at risk of psychological discomfort following contact with their GP *if* a positive test for type 2 diabetes is discovered after follow up with their GP.** Diabetes Australia state on their website that it ‘can be overwhelming’ to be diagnosed with diabetes as a result of learning that they have type 2 diabetes. An individual’s risk score is not a definitive diagnosis and as per current practice with completion of the AUSDRISK via the Diabetes Queensland website, all study participants who obtain an intermediate or high risk score will be advised to contact their GP to seek follow up advice (and possibly additional testing as deemed necessary by their GP). **Participants in this study will not receive a diagnosis of type 2 diabetes from the study activities per se** – they must receive this from their GP after further testing. Evidence suggests that the benefits of early detection of type 2 diabetes and subsequent long term impact of health and wellbeing far outweigh the emotional discomfort that may be encountered by any participants’ acknowledgement of their risk.

Please identify who (participants and/or others) the risk may affect.

Study participants

Please outline the mechanisms taken to minimise the risk.

1. All potential study participants will be provided with study information that includes the time expected for completion of each survey and as study participants will voluntarily commit to participate in the study, the likelihood of being inconvenienced will be low.
2. All study participants will be provided with study information that includes information about follow-up testing

for type 2 diabetes with their GP (what it involves and why it needs to happen)(this is current practice following online completion of the AUSDRISK with Diabetes Queensland). This letter will include study information for the participants GP's to understand the participants referral pathway (i.e. the study and process by which the participant has been recommended for follow-up testing for type 2 diabetes). This aims to ensure that both the participant and GP are informed about study processes and provides opportunity for GP's to give their patients further explanation and counselling regarding any subsequent diagnosis with type 2 diabetes (as required and as would normally occur if individuals are identified with intermediate or high risk of T2DM).

3. All study participants will also be provided with links to information available from professional diabetes organisations including Diabetes Queensland and Diabetes Australia – both of these organisations provide online information about 'living with diabetes' and include advice about 'what they can do if they have just been diagnosed'.

Importantly, information provided to the participants via video and text-based messages used in the study will include explanation of the potential benefits of early diagnosis that may occur through completion of the AUSDRISK and subsequent testing with their GP.

The overall likelihood of these risks for study participants is low and the potential benefits of this research to both the participant and the broader community, exceed the level of psychological risk posed to study participants.

Please indicate the potential benefits of the research.

Study participants:

Study participants who complete this study may benefit from early detection and/or diagnosis of type 2 diabetes. This early detection and/or diagnosis is known to delay and prevent onset of long-term complications, increase life expectancy and reduce lifetime costs.

Australian community:

This proof-of-concept study may provide evidence for the effectiveness of video-based storytelling in disease risk assessment and subsequently benefit many Australian adults in the future.

To whom the benefits are likely to accrue.

Direct benefits will be to Australian adults participating in the study.

If proof-of-concept is established from this study, the benefits could be seen in larger population groups (nationally) at risk of developing type 2 diabetes (currently ~2 million adults).

Researchers and public health practitioners will also benefit from any understanding gained during conduct of the study in relation to the impact of video-based storytelling in health promotion practice.

2.8 Location:

Is this research being conducted overseas ?

☐ YES ☒ NO If no, proceed to Part 3, Funding and Finance

If yes, In which country (or countries) is the research conducted ?

Describe the procedures by which overseas participants can obtain further information or complain about the research project?

On what basis is the research lawful in the jurisdiction(s) where it is to be conducted?

PART 3 FUNDING AND FINANCE

Researchers should include any source of funding (e.g., departmental, commercial, non-commercial, government) – National Statement on Ethical Conduct in Human Research 2007, Chapter 5.4.

3.1 Has this protocol received research funding or is this submission being made as part of an application for research funding?

☒ YES ☐ NO

3.2 What is the source of funding and has the funding been approved?

☒ YES ☐ NO

CQUniversity Merit Grant Scheme 2016/2017 – funding approved December 2016.

3.3 Will the researcher receive any remuneration and/or in kind funding to perform this research?

☐ YES ☒ NO

If yes, please provide details:

3.4 Will participants receive any payment or expenses for participation in the research?

☒ YES ☐ NO

If yes, please provide details:

As an incentive for participation across the 3 month period, all participants will be offered a $20 shopping voucher following completion of each of their one and three month follow-up surveys.

PART 4 OTHER APPROVALS

The principal researcher is responsible for informing each HREC of all other Australian sites at which the research is being proposed or conducted, at the time of submission of the research project, of any previous decisions regarding the research made by another HREC; and informing each HREC of whether the protocol is presently before another HREC (National Statement, Chapter 5.3).

4.1 Is this protocol being submitted or has it been previously submitted to another ethics committee?

☐ YES ☒ NO

4.2 If yes, give details of other centres involved; the approval status of the study at each centre; and details of any required amendments.

4.3 Other external approvals/reviews?

If your research has undergone peer review, review from a funding body or involves participants from other organisations, copies of letters of approval or reviews must be attached to this application (if pending at the time the application is submitted, forward to Ethics Officer when available). In some cases, institutions/authorities may decline to provide approval letters until ethics approval has been granted. In such cases, you should submit your application to the HREC for provisional approval pending receipt of the documentation.

4.4 Has the research undergone peer review, review from a funding body or does it involve participants from other organisations?

☒ YES ☐ NO If yes, specify from whom and attach a copy.

CQUniversity Merit Grant review team

PART 5 RECRUITMENT OF PARTICIPANTS

5.1 Provide number, age range and source of participants. This explanation should also include how potential participants will be identified and how initial contact will be made. For CQUniversity staff recruiting students as participants, please note that approval to access students is required from either the Dean of School, or from the Provost (Higher Education Division), depending on whether you are involving students from one school, or from across the higher education division.

We will use online methods (Facebook sites, community websites and email systems) to recruit a sample of Australian adults aged 35 years or older (based on age at which T2DM risk increases) who have no known diagnosis with T2DM. Electronic study 'advertisements' will be distributed to a range of health professionals (diabetes educators, dietitians, health clinics) and organisations to promote participation in the study.

5.2 What is the proposed method of recruitment of participants?

In alignment with AUSDRISK scoring and to ensure individuals with intermediate to high risk of T2DM are recruited, all potential participants will be screened for gender, age, ethnicity, height, weight, waist circumference, family history of T2DM, previous diagnosis with high blood glucose levels and blood pressure medication. All participants will be additionally screened for their living location in Australia to ensure only adults who are residing in Australia are recruited to the study. All eligible participants will be directed to the intervention website and asked to complete the online diabetes risk assessment (AUSDRISK) and a baseline survey. Follow-up surveys will occur one and three months following completion of their initial assessment.

PART 6 CONSENT

The potential participants must be provided with information ***at their level of comprehension*** *about the purpose, methods, demands, risks, inconveniences, discomforts and possible outcomes of the research (including the likelihood and form of publication of research results).*

Informing participants: Participants Information Sheet and Consent Form

6.1 Will the research involve informed consent of participants?

☒ YES ☐ NO

6.2 If yes, how will informed consent be obtained/recorded? If no, please justify why consent will not be obtained.

All potential participants will be provided with study information prior to commencement of the survey and informed that completion and submission of the questionnaire constitutes their consent to participate. This information will be provided on the information sheet of the survey. They will also be informed that they have the right to withdraw at any time before completing any of the surveys.

PART 7 INFORMATION PROTECTION (Confidentiality, Data Storage, Security and Disposal)

Confidentiality:

7.1 If you have indicated that participants will be identifiable, or re-identifiable, please provide an explanation why this is necessary.

Participants in the study will be re-identifiable to facilitate follow-up surveys to be conducted at one and three months following initial survey completion.

Participants will be asked to provide phone, email and postal contact details to allow follow up contact to be made and shopping vouchers to be forwarded.

7.2 Explain what methods will be used to guarantee confidentiality/anonymity of participant data.

The study website will be password protected and only study participants will be able to view the video-based stories that are provided following participants completion of their interactive online AUSDRISK.

Data Storage and security:

7.3 Explain how and where data will be held, including any arrangements for data security during the project?

A research data management plan will be established for this study and submitted to the Research Division. Data generated during this study will be stored in accordance with CQUniversity policy. i.e. all data, materials and related records will be stored in electronic form in a secure location on a shared drive. An active copy of research data and materials will be retained on the active drive of the CI's local computer hard drive at CQUniversity offices.

7.4 Please outline how long the data will be kept?

All research data and materials will be retained for at least five years after the date of the last publication that arises from the study.

7.5 Will the data be disposed of at some point?

☒ YES ☐ NO

7.6 If yes, how will the data be disposed of? If no, why is the data to be retained, and how/where will it be stored.

Research data and materials will be disposed of as required after the minimum 5 year retention period as noted above and once written permission for destruction has been provided by the University.

PART 8 DISSEMINATION OF RESULTS

8.1 Explain when, how, where and to whom results will be disseminated, including whether participants will be provided with information on the findings or outcomes of the project.

Results of this study will be disseminated to the broader research/academic community via publication in peer-reviewed journal manuscripts and related conference presentations.

All participants will be provided with a statement of study outcomes that will be emailed to them once all data analysis is complete. All results will be aggregated and no identifying information will be included in this statement.

PART 9 DECLARATIONS

Signatures and undertakings:

Applicant/Principal Researchers (including students and supervisors where appropriate)

I/We certify that:

- All information is correct and complete as possible;
- I/We have had access to and read the NHMRC 'National Statement on Ethical Conduct in Human Research', (2007);
- The research will be conducted in accordance with the National Statement;
- I/We have consulted any relevant legislation and regulations, and the research will be conducted in accordance with these;
- I/We will immediately report to the HREC anything that might warrant review of the ethical approval of the research including:
  - Serious or unexpected adverse effects on participants
  - Proposed changes in the protocol; and
  - Unforseen events that might affect continued ethical acceptability of the project;
- I/We have attempted to identify all the risks related to the research that may arise in conducting this research and acknowledge my/our obligations and the rights of participants;
- I/We will not continue the research if ethical approval or site authorisation is withdrawn and will comply with any special conditions required by the HREC, including:
  - Conditions of approval stipulated by the HREC; and
  - Cooperate with monitoring requirements. At a minimum annual progress reports and a final report will be provided to the HREC
- I/We have the appropriate qualifications, experience and facilities to conduct the research set out in the attached application and to deal with any emergencies and contingencies related to the research that may arise.

Print Name	Signature	Date
Susan L Williams		18/10/2017
Kate Ames		16/10/2017
Celeste Lawson		18/10/2017
Sonia Saluja		18/10/2017

Corneel Vandelanotte		15/10/2017
